# Serum selenium and reduced mortality in middle-aged and older adults with prefrailty or frailty: the mediating role of inflammatory status

**DOI:** 10.3389/fnut.2025.1560167

**Published:** 2025-07-28

**Authors:** Chih-Po Chang, Ching-Hui You

**Affiliations:** ^1^Division of Geriatric Medicine, Department of Internal Medicine, Mackay Memorial Hospital, Taipei City, Taiwan; ^2^Chu’s Family Medicine Clinic, Taipei City, Taiwan

**Keywords:** selenium, frailty, mortality, inflammation, National Health and Nutrition Examination Survey (NHANES)

## Abstract

**Background:**

Frailty is associated with increased mortality risk among middle-aged and older adults. Selenium, a trace element with antioxidant properties, may play a role in reducing mortality by modulating inflammatory processes. This study aimed to investigate the association between serum selenium and mortality in individuals with prefrailty or frailty, with a specific focus on potential mediators.

**Methods:**

Data of participants of National Health and Nutrition Examination Survey (1988–1994 and 2011–2016) of the US, aged 40–79 years with serum selenium measurements and frailty-related assessments were analyzed. All-cause and cardiovascular disease (CVD) mortality were confirmed by the National Death Index death certificate data. Systematic inflammation status was surrogated by the systemic immune inflammation index. Multivariable Cox proportional hazards models, restricted cubic spline (RCS) analysis, and mediation analysis were used to assess the associations.

**Results:**

Among 1,454 participants, those in the second, third, and fourth quartiles of serum selenium had significantly lower risks of all-cause mortality compared to the lowest quartile. The highest selenium quartile also showed a reduced risk of cardiovascular disease (CVD) mortality. Non-linear analysis indicated a significant relationship between selenium levels and all-cause mortality. Mediation analysis did not reveal that the protective effects of selenium were mediated by, CVD, chronic kidney disease, diabetes mellitus, or systemic inflammation status [as surrogated by the systemic immune-inflammation index (SII)].

**Conclusion:**

Higher serum selenium levels are linked to a lower risk of all-cause and CVD mortality in individuals with prefrailty or frailty. These findings highlight the need for future studies to clarify the pathways through which selenium may reduce mortality in prefrail and frail populations, and to determine whether selenium supplementation could offer therapeutic benefits.

## Introduction

With increasing life expectancy and declining birth rates, population aging has accelerated, drawing attention to age-related health issues. Frailty and prefrailty are common in middle-aged and older adults and are associated with higher risks of falls, disability, hospitalization, and mortality (1). Moreover, previous studies in the US have shown that hospitalization costs for frail patients are more than twice those of non-frail patients (2). Frail patients undergoing the same surgical procedures incur significantly higher median surgery costs, with hospitalization expenses increasing by approximately 30% (3). As the global population continues to age, identifying modifiable factors that can reduce mortality risk and alleviate healthcare costs in these populations has become a priority in public health research (4).

Frailty and prefrailty in middle-aged and older adults result from cumulative physiological decline driven by biological aging, including chronic inflammation, hormonal dysregulation, and mitochondrial dysfunction (5). These intrinsic changes are compounded by sarcopenia, multimorbidity, and nutritional deficiencies, leading to diminished physical resilience. Physical inactivity and psychosocial stressors, such as depression and social isolation, further accelerate the loss of functional capacity (6). Gender differences and socioeconomic disparities also influence vulnerability to frailty through varied exposure to risk factors and access to healthcare. Therefore, frailty emerges as a multifactorial syndrome rooted in both biological deterioration and modifiable lifestyle and environmental factors (7).

Selenium is an essential trace element and incorporated into selenoproteins, like glutathione peroxidases (GPx), thioredoxin reductases, and selenoprotein P, which have critical antioxidant, detoxication, anti-inflammatory, immune-modulatory, and anti-cancer functions (8). The normal serum selenium levels in adults typically ranges from 120–160 (μg/l) (9). Clinical studies have demonstrated that lower serum selenium levels are significantly associated with decreased muscle strength, impaired physical performance, and higher frailty prevalence in aging populations (10, 11). Furthermore, selenium contributes to mitochondrial function and protein synthesis in skeletal muscle, thereby helping to counteract sarcopenia and physical decline (12).

Previous studies indicated that selenium may affect health outcomes by modulating inflammatory processes, which are frequently elevated in individuals with frailty and prefrailty (8, 13, 14). Inflammation is a well-established contributor to the progression of frailty, and elevated inflammatory markers have been associated with increased mortality in this population. Selenium’s anti-inflammatory effects may offer a protective mechanism against the adverse outcomes associated with frailty. However, the relationship between serum selenium levels, inflammatory status, and mortality in frail and prefrail individuals has not been thoroughly investigated (15).

This study aims to investigate the association between serum selenium and mortality in middle-aged and older adults with prefrailty or frailty, with a particular focus on the mediating role of inflammation, using data from a large, nationally representative sample from the National Health and Nutrition Examination Survey (NHANES) of the US.

## Methods

### Study design and data source

This population-based retrospective cohort study analyzed data from the publicly released two-year cycles of the US National Health and Nutrition Examination Survey (NHANES) database. Data from NHANES III (1988–1994) and NHANES 2011–2016 were used to performed the analysis.

NHANES data were collected by the National Center for Health Statistics (NCHS), a division of the Centers for Disease Control and Prevention (CDC) of the US. The survey is designed to evaluate the health and nutritional status of adults and children in the US. It uses a complex, multistage design to collect and analyze data representative of the national, non-institutionalized US population. The data are released for research purposes and permission to use the data is granted to researchers by the NCHS. Participants in NHANES complete a household interview and are invited for an extensive examination in a NHANES mobile examination center (MEC), including a physical examination, specialized measurements, and laboratory tests. Consequently, an evaluation of participants in the NHANES database is reliable and multidimensional and can be equated to a population-level assessment (16, 17).

### Ethics statement

The NCHS Research Ethics Review Board reviewed and approved NHANES, and all survey participants provided signed informed consent to participate. Therefore, no further ethical approval and informed consent were required to perform the secondary analyses undertaken in this study. Additionally, all NHANES data released by the NCHS are de-identified and all patient data remain anonymous during data analysis.

### Study population selection

This study included data from community-dwelling adults aged 40–79 years, collected during the 1988–1994 (NHANES III) and 2011–2016 (NHANES) periods. These data cycles were selected due to the availability of serum selenium measurements and the information needed to assess frailty status. Participants were excluded if they were not classified as frail or prefrail, had cancer at baseline, or had no data on follow-up all-cause or cardiovascular deaths, sex, or sample weights.

### Assessment of prefrailty and frailty

Frailty status was assessed using the Fried Frailty Phenotype (FP). The Fried Frailty Phenotype evaluates physical frailty based on five criteria: unintentional weight loss; weakness or poor handgrip strength; self-reported exhaustion; slow walking speed; and low physical activity (18). For this study, we used an adapted version of the Fried Frailty Phenotype, as modified in previous NHANES studies (19). This modified version is an acceptable alternative for large-scale research when direct physical measurement are unavailable. It include four criteria: (1) unintentional weight loss; (2) exhaustion; (3) weakness; and (4) low physical activity. Participants who scored one point were classified as pre-frail, while those scoring two points or more were classified as frail.

### Serum selenium levels

The laboratory procedures and quality control methods for serum selenium measurements in NHANES III and NHANES 2011–2016 have been well-documented (NHANES III: https://stacks.cdc.gov/view/cdc/45776, NHANES 2011–2012: https://wwwn.cdc.gov/nchs/nhanes/continuousnhanes/manuals.aspx?BeginYear=2011, NHANES 2013–2014: https://wwwn.cdc.gov/nchs/nhanes/continuousnhanes/manuals.aspx?BeginYear=2013, and NHANES 2015–2016: https://wwwn.cdc.gov/nchs/nhanes/continuousnhanes/manuals.aspx?BeginYear=2015). In NHANES III, atomic absorption spectrometry (AAS) was used to quantify serum selenium, while Inductively Coupled Plasma Dynamic Reaction Cell Mass Spectrometry (ICP-DRC-MS) was utilized for the NHANES 2011–2016 measurements. All selenium levels were standardized to μg/L for analysis. For NHANES 2011–2016, the data was derived from a one-third random sample.

### All-cause mortality and CVD mortality

Throughout the end of 2019, the NHANES participants were linked to the National Death Index (NDI) death certificate data which contains nine-cause specific death categories. This enabled further ascertainment of both the mortality status and the underlying leading cause of death. All-cause mortality was defined as deaths from any cause, and was the main study endpoint in this investigation. CVD mortality was defined separately as deaths from cardiac and cerebrovascular disease through codes: UCOD_LEADING = 001 (‘disease of the heart’) or 005 (‘cerebrovascular disease’). More information about the Linked Mortality Files and definition of cause of death are available at: https://wwwn.cdc.gov/nchs/nhanes/continuousnhanes/manuals.aspx?BeginYear=2011.

### Covariates

Demographic data, including age, sex, race, education level, and cigarette smoking were obtained through in-person interviews conducted by trained NHANES interviewers using the Family and Sample Person Demographics questionnaires and the Computer-Assisted Personal Interviewing (CAPI) system (Confirmit Corp. New York, United States). Collected data were weighted according to the NHANES protocol.

BMI was obtained from the NHANES examination measurements, calculated as body weight (kilograms) divided by height (meters squared). Hypertension was defined by those who responded “yes” to the questions: “Were you told on two or more different visits that you had hypertension, also called high blood pressure?” or “Because of your (high blood pressure/hypertension), have you ever been told to … take prescribed medicine?,” or with an average of three consecutive measures of systolic blood pressure ≥ 140 mmHg, or with an average of three consecutive measures of diastolic blood pressure ≥ 90 mmHg.

Diabetes mellitus (DM) were defined based on the following criteria, and participants meeting any of these conditions were excluded from the study cohort: a positive response to the questions: “Are you taking insulin?,” “Did a doctor tell you, you have diabetes?,” “Do you take pills to lower blood sugar?”; an HbA1c ≥ 6.5%; a fasting glucose level of ≥ 126 mg/dL; or a glucose level of ≥ 200 mg/dL during the oral glucose tolerance test (OGTT), as recorded in the NHANES laboratory data.

CVD history was defined based on responses to the following questions: whether a physician had ever diagnosed coronary heart disease, angina, heart attack, stroke, or congestive heart failure. Chronic respiratory disease was defined as the presence of any of the following conditions: asthma, chronic bronchitis, or emphysema. Chronic kidney disease (CKD) was determined by a GFR < 60 mL/min/1.73 m^2^. GFR was estimated from re-calibrated serum creatinine using the 4-variable Modification of Diet in Renal Disease (MDRD) Study equation.

Participants’ smoking status was classified into three categories: non-smoker, former smoker, or current smoker, based on the following criteria: non-smoker if they had smoked fewer than 100 cigarettes in their lifetime; former smoker if they had smoked more than 100 cigarettes in their lifetime but were not currently smoking; and current smoker if they had smoked more than 100 cigarettes in their lifetime and were still smoking.

Regarding the laboratory measures, the analysis included the white blood cell (WBC) count, neutrophil count, platelet count, and lymphocyte count. We also calculated the systemic immune inflammation index (SII) using these measures, with the formula as follows: *SII = Neutrophil count × Platelet count/Lymphocyte count*. SII was analyzed both as a continuous variable and as a categorical variable based on quartiles, with high SII defined as Quartile 4 and low SII defined as Quartile 1 through Quartile 3.

### Statistical analysis

The NHANES employs a complex, multistage probability sampling design to ensure national representation. To account for this design, we applied sampling weights (WTMEC2YR, WTPFHSD6), pseudo-stratum (SDMVSTRA, SDPSTRA6), and pseudo-cluster (SDMVPSU, SDPPSU6) variables in all analyses, following the NCHS guidelines. Analyses were conducted using the SURVEY procedure in SAS. Descriptive statistics are presented as numbers (n) and weighted percentages (%), or as means with standard errors (SE). Group comparisons were conducted using the Rao-Scott chi-square test for categorical variables and linear models for continuous variables.

Multivariable Cox proportional hazards models were used to assess the association between serum selenium levels and mortality, adjusting for variables with a *p*-value < 0.05 in univariable analyses, excluding laboratory measures and frailty status.

Restricted cubic spline (RCS) models with three knots (at the 25th, 50th, and 75th percentiles) were applied to evaluate the relationship between serum selenium levels and all-cause mortality as well as CVD mortality. Additionally, mediation analysis was conducted to explore whether the association between serum selenium levels and all-cause mortality was mediated by CVD, DM, CKD, and SII. Statistical significance was determined with a two-sided *p*-value of <0.05. All analyses were conducted using SAS statistical software (version 9.4, SAS Inc., Cary, NC, United States).

## Results

### Study population

**Figure 1:**
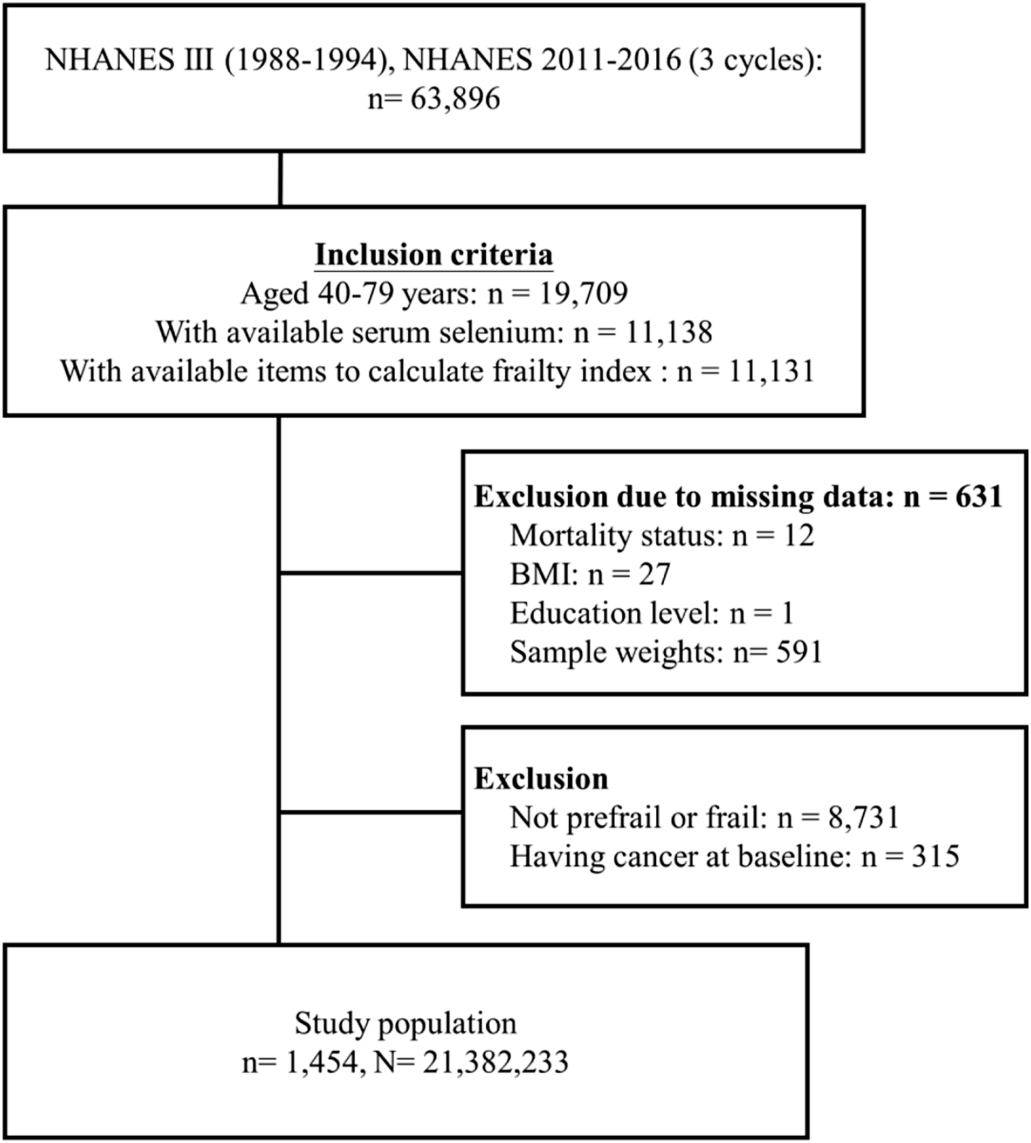
Flow diagram of study cohort selection (*n* refers to a sample size while *N* refers to the population size).

A total of 63,896 participants were collected from the NHANES III (1988–1994), and three study cycles of the NHANES (2011–2012, 2013–2014, and 2015–2016). From this group, 11,131 individuals aged 40–79 years with available serum selenium levels and frailty index data were included. We excluded 631 participants due to missing information on mortality status, BMI, education level, or weight. Additionally, 8,731 participants who were neither prefrail nor frail, as well as 315 individuals with a cancer diagnosis at baseline, were excluded. Ultimately, 1,454 participants with prefrailty or frailty were selected for further analyses. After weighting, this sample was representative of an estimated 21,382,233 US residents (Figure 1).

### Characteristics of the individuals with prefrailty or frailty

Table 1 presents the characteristics of the study population, divided into three groups based on quartiles of serum selenium levels. The mean age was 58.9 years, with 60.5% being female. Non-Hispanic White participants made up the majority of the study population, accounting for 73.1%. All-cause mortality rates differed significantly among the groups, being highest in the lowest quartile (Q1) and decreasing as selenium levels increased (*p* = 0.014). Although cardiovascular disease mortality followed a similar trend, it did not reach statistical significance. Race, prevalence of diabetes mellitus (DM), cardiovascular disease (CVD), chronic kidney disease (CKD), neutrophil counts, and SII levels also varied significantly across the selenium quartiles, and these variables will be accounted for in the subsequent multivariable analysis (Table 1).

**Table 1:** Characteristics of the study population according to serum selenium.

Characteristics	Total (*n* = 1,454)	Serum selenium, ug/L				
		Quartile 1 (≤114.9)*n* = 363	Quartile 2 (114.9–125.1)*n* = 364	Quartile 3 (125.1–136.9)*n* = 361	Quartile 4 (>136.9)*n* = 366	*p*-value
Outcome						
All-cause mortality	509 (35.3)	158 (47.5)	154 (35.7)	100 (32.0)	97 (28.8)	**0.014**
Cardiovascular mortality	193 (12.8)	56 (17.7)	55 (13.3)	47 (12.7)	35 (8.6)	0.069
Demography						
Frailty						0.067
Pre-frailty	1,118 (81.6)	261 (73.5)	280 (81.8)	289 (84.5)	288 (84.8)	
Frailty	336 (18.4)	102 (26.5)	84 (18.2)	72 (15.5)	78 (15.2)	
Age, years	58.9 ± 0.5	60.2 ± 0.8	58.1 ± 0.9	58.5 ± 0.7	58.9 ± 0.6	0.310
40–49	291 (25.8)	67 (19.4)	66 (27.1)	82 (29.0)	76 (26.5)	0.419
50–59	312 (22.8)	78 (24.8)	69 (22.9)	71 (19.1)	94 (24.6)	
60–69	522 (31.6)	132 (32.8)	151 (33.9)	130 (33.7)	109 (26.6)	
70–79	329 (19.8)	86 (22.9)	78 (16.0)	78 (18.2)	87 (22.3)	
Sex						0.218
Male	590 (39.5)	140 (36.0)	141 (34.4)	150 (41.9)	159 (44.2)	
Female	864 (60.5)	223 (64.0)	223 (65.6)	211 (58.1)	207 (55.8)	
Race						**<0.001**
Non-hispanic White	545 (73.1)	119 (65.0)	124 (72.7)	138 (73.4)	164 (79.3)	
Non-hispanic Black	384 (11.8)	126 (19.0)	107 (13.5)	90 (9.8)	61 (6.9)	
Mexican American	267 (4.9)	67 (5.7)	75 (4.9)	62 (4.2)	63 (4.9)	
Others	258 (10.2)	51 (10.3)	58 (9.0)	71 (12.6)	78 (8.9)	
BMI, kg/m^2^	29.6 ± 0.3	29.6 ± 0.4	28.8 ± 0.5	30.1 ± 0.5	29.7 ± 0.5	0.440
Obesity	647 (41.6)	177 (44.4)	152 (38.5)	158 (44.0)	160 (39.7)	0.576
Education level						0.098
High school or above	768 (39.3)	206 (47.2)	202 (37.8)	175 (34.3)	185 (39.5)	
Below high school	686 (60.7)	157 (52.8)	162 (62.2)	186 (65.7)	181 (60.5)	
Cigarette smoking						0.073
Never	672 (45.0)	161 (42.3)	174 (47.1)	164 (43.9)	173 (46.4)	
Former	437 (32.3)	107 (29.4)	94 (26.2)	115 (36.1)	121 (36.1)	
Current	345 (22.7)	95 (28.3)	96 (26.7)	82 (20.0)	72 (17.4)	
Hypertension	873 (53.8)	221 (56.8)	213 (50.4)	214 (48.7)	225 (59.4)	0.194
Chronic respiratory disease	304 (22.5)	72 (22.8)	75 (18.3)	71 (22.7)	86 (25.7)	0.328
DM	466 (25.2)	115 (27.0)	124 (26.8)	93 (17.7)	134 (29.9)	**0.030**
CVD	315 (16.5)	97 (21.3)	67 (11.7)	66 (14.5)	85 (19.0)	**0.040**
CKD	363 (25.1)	107 (33.2)	104 (26.0)	77 (23.1)	75 (20.2)	**0.030**
Laboratory measures						
WBC count, 10^9^/L (Missing = 2)	428.2 ± 320.0	40.2 ± 24.7	426.3 ± 25.5	548.8 ± 544.1	607.0 ± 581.1	0.535
Neutrophil count, 10^9^/L (Missing = 349)	4.5 ± 0.1	4.4 ± 0.2	4.3 ± 0.1	4.5 ± 0.2	4.7 ± 0.2	**<0.001**
Platelet count, 10^9^/L (Missing = 2)	669.3 ± 319.1	274.8 ± 25.1	671.5 ± 27.5	792.9 ± 542.3	846.7 ± 579.8	0.521
Lymphocyte count, 10^9^/L (Missing = 3)	423.1 ± 320.1	35.1 ± 24.8	421.3 ± 25.5	543.8 ± 544.2	601.7 ± 581.2	0.535
SII, 10^9^/L^a^	571.7 ± 16.5	587.7 ± 28.2	518.1 ± 17.8	584.5 ± 29.1	589.1 ± 24.4	**<0.001**
High (>500)	275 (26.4)	58 (27.5)	61 (22.2)	68 (25.3)	88 (29.8)	0.428
Low (≤500)	830 (73.6)	206 (72.5)	183 (77.8)	226 (74.7)	215 (70.2)	
Missing	349	99	120	67	63	

SII, Systemic immune-inflammatory index; BMI, body mass index; DM, diabetes mellitus; CKD, chronic kidney disease; CKD, chronic kidney disease; WBC, white blood cell. ^a^A high SII was defined as the highest quartile (Q4), while a low SII was defined as quartiles Q1 to Q3. Continuous variables are presented as mean ± SE. Categorical variables are presented as unweighted counts (weighted percentages). *p*-values < 0.05 are shown in bold.

### Association between serum selenium and mortality in individuals with prefrailty and frailty

Table 2 shows the associations between serum selenium levels, all-cause mortality, and cardiovascular disease (CVD) mortality in individuals with prefrailty and frailty. After adjusting for relevant confounders in the multivariable analysis, participants in the second quartile of serum selenium (aOR = 0.61, 95% CI: 0.44–0.84, *p* = 0.003), third quartile (aOR = 0.70, 95% CI: 0.51–0.96, *p* = 0.026), and fourth quartile (aOR = 0.59, 95% CI: 0.36–0.99, *p* = 0.048) had a significantly lower risk of all-cause mortality. Additionally, participants in the fourth quartile had a significantly reduced risk of CVD mortality (aOR = 0.42, 95% CI: 0.19–0.92, *p* = 0.031) (Table 2).

**Table 2:** Associations between serum selenium levels, all-cause mortality, and CVD mortality.

Multivariable analysis	All-cause mortality		CVD mortality	
	aHR^a^ (95% CI)	*p*-value	aHR^b^ (95% CI)	*p*-value
Serum selenium, ug/L	0.99 (0.98–1.01)	0.360	0.99 (0.97–1.00)	0.120
Q1	Ref.		Ref.	
Q2	**0.61 (0.44–0.84)**	**0.003**	0.67 (0.39–1.16)	0.151
Q3	**0.70 (0.51–0.96)**	**0.026**	0.78 (0.45–1.36)	0.384
Q4	**0.59 (0.36–0.99)**	**0.048**	**0.42 (0.19–0.92)**	**0.031**

HR, hazard ratio; aHR, adjusted hazard ratio; CI, confidence interval; ref, reference; CVD, cardiovascular disease. *P*-values < 0.05 are shown in bold. ^a^Adjusted for variables with a *p*-value < 0.05 in the univariable analysis, excluding lab measures and frailty, including age (in years), education, smoking, hypertension, DM, CVD, and CKD. ^b^Adjusted for variables with a *p*-value < 0.05 in the univariable analysis, excluding lab measures and frailty, including age (in years), education, cigarette smoking, DM, CVD, and CKD.

### Restricted cubic spline analysis

**Figure 2:**
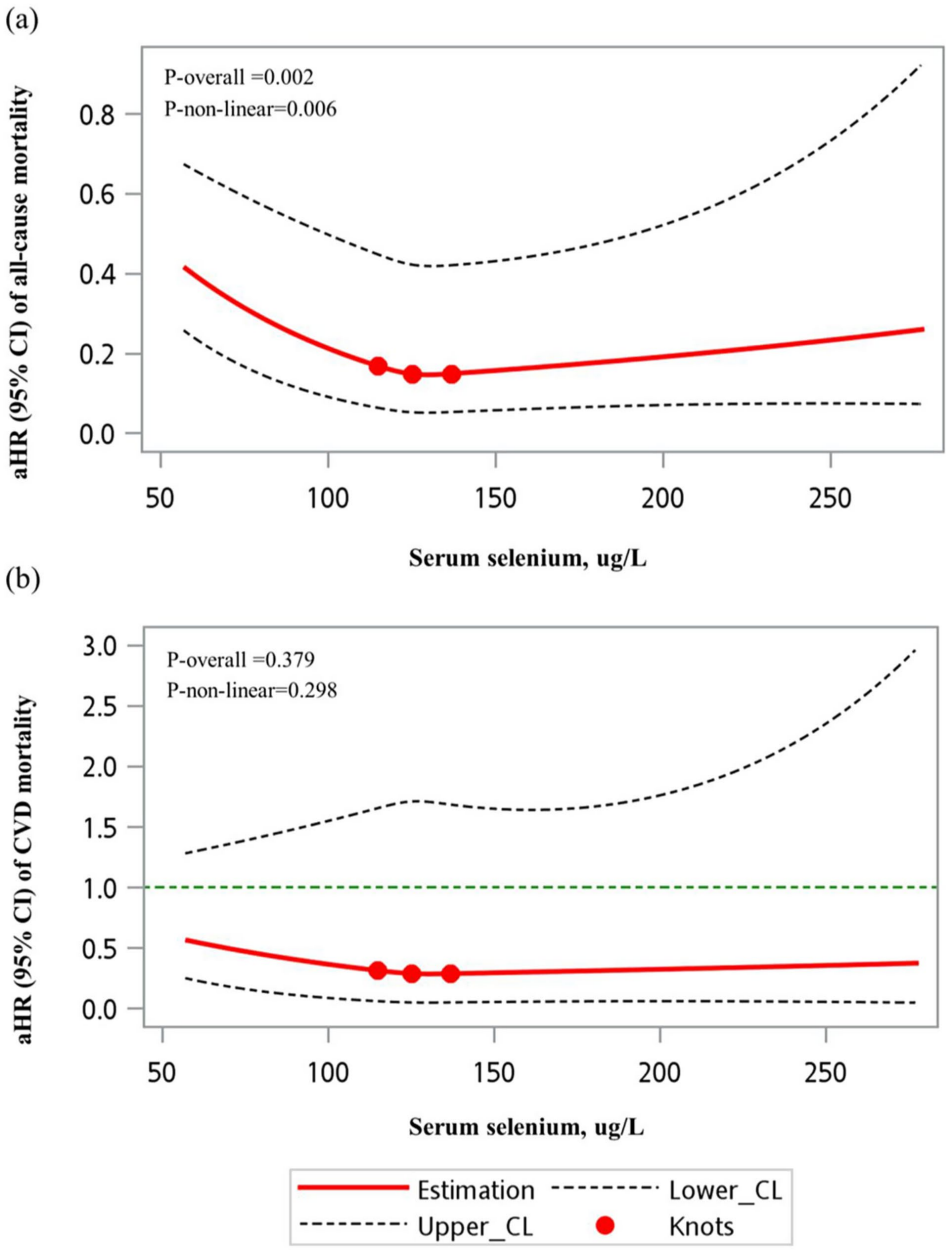
RCS models of the association between serum selenium (unweighted) and mortality outcomes. **(a)** All-cause mortality and **(b)** cardiovascular mortality. A significant non-linear association was observed between serum selenium levels and all-cause mortality (*p* for non-linearity = 0.006). However, no significant non-linear relationship was found for cardiovascular mortality (*p* for non-linearity = 0.297).

The restricted cubic spline (RCS) model revealed a significant non-linear association between selenium levels and all-cause mortality risk, as shown in Figure 2 (*p*-overall = 0.002, *p* for non-linearity = 0.006). For CVD mortality, although a similar trend was observed, it did not reach statistical significance (*p*-overall = 0.379, *p* for non-linearity = 0.298) (Figure 2).

### Mediation analysis

**Figure 3:**
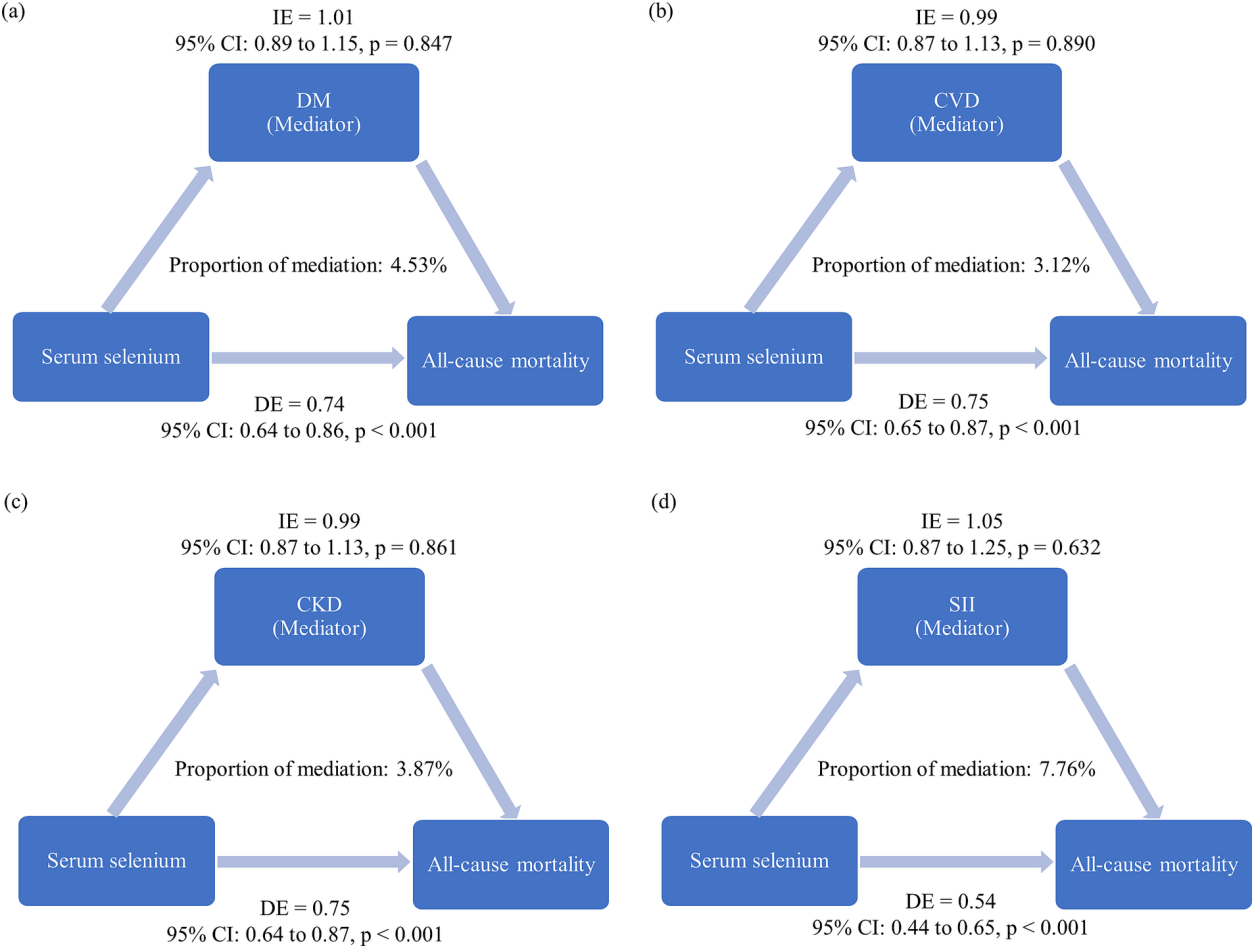
Mediation analysis results. The estimated proportion of the association between serum selenium levels (quartile 4 vs. quartile 1) and all-cause mortality mediated by: **(a)** Diabetes mellitus (DM), **(b)** cardiovascular disease (CVD), **(c)** chronic kidney disease (CKD), and **(d)** systemic inflammation index (SII). The model was adjusted for variables with *p* < 0.05 in the univariable analysis, excluding laboratory measures, frailty, DM, CVD, and CKD. Covariates included age (in years), education level, cigarette smoking, and hypertension. IE: Adjusted hazard ratio for the indirect effect. DE: Adjusted hazard ratio for the direct effect. Proportion of mediation: Calculated as log (IE)/log (DE + IE).

We further conducted mediation analysis to evaluate whether the protective association between a high serum selenium level (quartile 4 vs. quartile 1) and all-cause mortality was mediated through chronic disease including DM, CVD, and CKD, as well as body’s systemic inflammation status (surrogated by SII). The results revealed that while selenium had a significant direct effect on all-cause mortality (*p* < 0.001), the mediation effects through DM, CVD, CKD, or systemic inflammation status did not reach statistical significance (*p* > 0.05) (Figures 3a–d).

## Discussion

In this study, using data from the NHANES, we investigated the association between serum selenium levels and mortality in the prefrail and frail elderly population. We found that, after adjusting for confounding factors, participants with higher serum selenium levels showed a significantly reduced risk of all-cause mortality, with reductions of up to approximately 40%. Additionally, those in the highest quartile of serum selenium experienced about a 60% lower risk of CVD mortality compared to the lowest quartile.

Frailty is a clinical syndrome marked by increased vulnerability to adverse health outcomes due to declining physiological reserves across multiple systems. Frail individuals are more prone to stressors like infections or surgeries, leading to worse outcomes such as falls, hospitalization, and disability (20). Though commonly linked to aging, it can occur at any adult stage. While more prevalent in older adults, frailty affects 4–10% of middle-aged individuals (45–64 years), with higher rates seen in those with chronic disease, obesity, or lower socioeconomic status (21). On the other hand, proper nutrition has been shown to help reverse frailty by providing essential nutrients such as proteins, vitamins (D, B12), and minerals, all of which support muscle strength, bone health, and overall physical function (22, 23).

Current evidence suggests that selenium levels are closely associated with mortality risk across different populations. A meta-analysis by Xiang et al. (14) found that low circulating selenium was linked to a higher risk of cardiovascular and all-cause mortality in the general population, with pooled risk ratios of 1.36 and 1.35, respectively. This highlights the detrimental effects of selenium deficiency on survival outcomes. In individuals with type 2 DM, a study by Qiu et al. (24) demonstrated that higher selenium levels were significantly associated with reduced risks of both all-cause and heart disease mortality, with the highest quartile of selenium concentration showing a 31% lower risk of all-cause mortality. Similarly, Zhu et al. (25) reported that in CKD patients, higher serum selenium levels were associated with a lower risk of all-cause and CVD mortality, with hazard ratios of 0.684 and 0.513, respectively, for the highest quartiles. Similarly, a study by Anadón Ruiz et al. (26) in end-stage renal disease (ESRD) patients on dialysis found that low plasma selenium levels (<63 μg/L) were linked to a nearly threefold increase in the risk of all-cause mortality, independent of other clinical markers. Collectively, these studies underscore the importance of maintaining adequate selenium levels for improving survival outcomes across different populations and conditions, reinforcing selenium’s role as a potential biomarker for mortality risk. While no studies have specifically focused on the prefrail and frail populations, making direct comparisons difficult, our study indicates that the reduced mortality risk associated with higher selenium levels in these groups is even greater than that observed in the DM population and is comparable to the effect size seen in CKD patients. This underscores the potential importance of selenium for vulnerable populations.

Beyond its potential impact on frailty, selenium has been studied for its role in managing other inflammatory diseases. For instance, a randomized, double-blind, placebo-controlled clinical trial conducted by Khazdouz et al. investigated selenium supplementation in patients with mild-to-moderate ulcerative colitis, an inflammatory bowel disease. The study reported that selenium administration (oral selenomethionine capsule, 200 mcg/day) led to clinical improvement and remission, highlighting its anti-inflammatory properties in gastrointestinal disorders (27). In the context of rheumatoid arthritis, a randomized clinical trial implemented by Zamani et al. found that selenium supplementation (200 mcg/day) resulted in a modest but clinically relevant improvement in rheumatoid arthritis biomarkers such as erythrocyte sedimentation rate (ESR) and anti-cyclic citrullinated peptide (anti-CCP) antibodies. This suggests a potential role for selenium in modulating inflammatory responses in autoimmune conditions (28). Furthermore, a review research by Barchielli illustrated selenium’s involvement in various pathologies, including its anti-inflammatory effects in conditions like Hashimoto’s thyroiditis and other autoimmune diseases. The review emphasized selenium’s capacity to reduce oxidative stress and modulate immune responses, contributing to its therapeutic potential in inflammatory disorders (29). These findings underscore selenium’s broader significance in managing inflammatory diseases, reinforcing the importance of adequate selenium intake for overall health and disease prevention.

On the other hand, while observational studies suggest a strong association between selenium levels and reduced mortality risk, findings from clinical trials offer a less optimistic view. A systematic review and meta-analysis of randomized controlled trials (RCTs) by Jenkins et al. (13) found no significant effect of selenium supplementation alone on cardiovascular or all-cause mortality. The dosage of selenium from included RCTs ranged from 50 to 200 mcg/day, regardless of other supplement exposure like vitamin C, zinc, etc. The benefits were only observed when selenium was included as part of antioxidant mixtures, which showed a modest reduction in mortality risk. This contrast between the strong associations in observational studies and the more limited effects seen in clinical trials suggests that the protective role of selenium might be more complex, possibly influenced by interactions with other nutrients or factors that observational designs may not fully account for. Besides, the Nutritional Prevention of Cancer (NPC) RCT-designed study administered 200 micrograms of selenium daily to participants and observed a significant reduction in total cancer incidence. However, increasing the dose to 400 mcg per day did not confer additional benefits, suggesting a plateau effect at the 200 mcg dosage (30). The NPC study reported a decrease in lung, colon, and prostate cancers with 200 mcg of daily selenium supplementation. However, there was an observed increase in nonmelanoma skin cancer risk, highlighting the complexity of selenium’s effects on different cancer types (30). Moreover, cisplatin, a platinum-based chemotherapy used for various cancers, lowers selenium levels in hair and serum, though the clinical relevance remains unclear (31, 32). Small studies suggest selenium may reduce cisplatin-induced toxicity (33), but a Cochrane Review found insufficient evidence to confirm its benefit in mitigating chemotherapy side effects (34).

Selenium supplementation is typically safe and well-tolerated when maintained within recommended dosages. The tolerable upper intake level (UL) for selenium, established at 400 mcg per day for adults, serves as a critical threshold to prevent adverse effects (35). Excessive intake beyond this limit has been associated with selenosis, a condition marked by a spectrum of symptoms including gastrointestinal discomfort, alopecia, brittle nails, and neurological impairments (35, 36).

Importantly, previous studies have not clarified whether the protective effect of higher selenium levels on mortality is mediated by factors such as chronic diseases like CVD, DM, or CKD. Our study additionally evaluated these potential mediating mechanisms; however, the findings did not demonstrate statistically significant mediation effects of CVD, DM, CKD, or inflammation status (as indicated by SII) on the association between higher selenium levels and reduced mortality. Nevertheless, human studies in the literature have shown that individuals with higher selenium levels have lower markers of systemic inflammation, including CRP, IL-6, and TNF-*α*. For example, A very recent meta-analysis demonstrated that intravenous selenium supplementation significantly reduces CRP and IL-6 plasma concentrations. However, it also showed an unexpected increase in TNF-α levels (37). Another recent study reported that selenium supplementation can have promising effects by alterations of inflammatory markers for critically ill patients (38).

Previous studies exploring underlying biological mechanisms may offer additional insights. Selenium’s role in reducing chronic inflammation, a key driver of frailty and mortality, is supported by experimental evidence. Selenoproteins, such as glutathione peroxidases (GPXs) and thioredoxin reductases (TXNRDs), are integral to neutralizing oxidative stress and regulating inflammatory pathways (39). In animal models, selenoprotein deficiency leads to increased susceptibility to inflammation and oxidative stress, which can drive chronic conditions like atherosclerosis and insulin resistance—key comorbidities of frailty (29). Cell experiments also support this mechanism. In macrophage cell lines, selenium supplementation has been shown to inhibit the NF-κB signaling pathway, a key regulator of pro-inflammatory gene expression, by enhancing the activity of selenoproteins like GPXs. The NF-κB pathway is responsible for the transcription of pro-inflammatory cytokines, and its inhibition by selenium leads to a reduction in cytokine production and inflammation (40).

In summary, the interplay between selenium, inflammation, chronic disease, frailty, and mortality may be more complex than previously understood or than initially hypothesized. This emphasizes the need for further mechanistic and prospective studies to clarify how selenium’s anti-oxidative and anti-inflammatory effects contribute to improved long-term survival in pre-frail and frail populations.

### Clinical implications

Clinically, selenium status may serve as a potential biomarker for mortality risk in pre-frail and frail populations. Routine selenium supplementation should be approached cautiously until further evidence from interventional studies becomes available.

### Future direction

Given the significant association identified between higher serum selenium levels and reduced all-cause and cardiovascular mortality, future studies should prioritize confirming these findings through well-designed prospective and interventional trials. Specifically, RCTs in prefrail and frail populations are warranted to determine the causal impact of selenium supplementation on mortality and functional outcomes. Additionally, research should explore optimal selenium dosing strategies, considering baseline nutritional status, age, sex, comorbidities, and genetic polymorphisms in selenoprotein expression. Mechanistic studies are also needed to elucidate the molecular pathways through which selenium modulates inflammatory responses and oxidative stress in frail states. Moreover, longitudinal analyses capturing dynamic changes in selenium levels, inflammatory biomarkers, and frailty progression could provide valuable insights into the temporal relationship and therapeutic windows for intervention. Finally, given global dietary variations, population-specific research is essential to tailor selenium-based strategies to diverse nutritional and healthcare contexts.

### Strengths and limitations

This study has several strengths. It uses data from the NHANES, which provides a large, nationally representative sample of the U.S. population. The inclusion of participants from two NHANES cycles (NHANES III and NHANES 2011–2016) allows for a comprehensive analysis across different time periods, increasing the generalizability of the findings. Additionally, the study employs rigorous statistical methods, including multivariable Cox proportional hazards models, restricted cubic spline analysis, and mediation analysis, to explore the complex relationship between serum selenium, inflammation, and mortality. By examining a wide range of covariates, such as demographic data, lifestyle factors, and health conditions, the analysis controls for potential confounding variables, enhancing the validity of the results. The study also uses a standardized and well-validated method to assess frailty status, allowing for accurate classification of participants into prefrail and frail groups.

However, the study has some limitations. The observational design limits the ability to establish causal relationships between serum selenium levels and mortality. Although mediation analysis was used to explore the role of inflammation, other unmeasured factors, such as dietary habits, genetic variations, or long-term lifestyle changes, might influence both selenium levels and mortality risk. Additionally, serum selenium was measured only once for each participant, which may not fully capture long-term selenium status. The use of a modified Fried Frailty Phenotype, adapted for large-scale research, may not be as precise as direct physical measurements. Lastly, the study is based on data from U.S. adults, which may limit the applicability of the findings to populations with different dietary selenium intake or genetic backgrounds. Despite these limitations, this study provides valuable insights into the potential role of selenium in mortality risk among frail and prefrail individuals.

## Conclusion

In conclusion, this study demonstrates that high serum selenium levels are independently associated with a reduced risk of CVD mortality and all-cause mortality in middle-aged and older adults with prefrailty or frailty. Importantly, our findings also identify systemic inflammation as a predominant mediator in this association. While our findings underscore the relevance of serum selenium in mortality risk, further research, particularly interventional studies, is necessary to clarify causal mechanisms and evaluate the potential role of selenium in public health strategies targeting aging and frail populations.

## Data Availability

The original contributions presented in the study are included in the article/supplementary material, further inquiries can be directed to the corresponding author.
